# decoupleR: ensemble of computational methods to infer biological activities from omics data

**DOI:** 10.1093/bioadv/vbac016

**Published:** 2022-03-08

**Authors:** Pau Badia-i-Mompel, Jesús Vélez Santiago, Jana Braunger, Celina Geiss, Daniel Dimitrov, Sophia Müller-Dott, Petr Taus, Aurelien Dugourd, Christian H Holland, Ricardo O Ramirez Flores, Julio Saez-Rodriguez

**Affiliations:** 1 Heidelberg University, Faculty of Medicine, and Heidelberg University Hospital, Institute for Computational Biomedicine, BioQuant, Heidelberg 69120, Germany; 2 Institute for Computational Biomedicine, Heidelberg University Hospital, BioQuant, Heidelberg 69120, Germany; 3 Central European Institute of Technology, Masaryk University, Brno 601, Czechia

## Abstract

**Summary:**

Many methods allow us to extract biological activities from omics data using information from prior knowledge resources, reducing the dimensionality for increased statistical power and better interpretability. Here, we present decoupleR, a Bioconductor and Python package containing computational methods to extract these activities within a unified framework. decoupleR allows us to flexibly run any method with a given resource, including methods that leverage mode of regulation and weights of interactions, which are not present in other frameworks. Moreover, it leverages OmniPath, a meta-resource comprising over 100 databases of prior knowledge. Using decoupleR, we evaluated the performance of methods on transcriptomic and phospho-proteomic perturbation experiments. Our findings suggest that simple linear models and the consensus score across top methods perform better than other methods at predicting perturbed regulators.

**Availability and implementation:**

decoupleR’s open-source code is available in Bioconductor (https://www.bioconductor.org/packages/release/bioc/html/decoupleR.html) for R and in GitHub (https://github.com/saezlab/decoupler-py) for Python. The code to reproduce the results is in GitHub (https://github.com/saezlab/decoupleR_manuscript) and the data in Zenodo (https://zenodo.org/record/5645208).

**Supplementary information:**

Supplementary data are available at *Bioinformatics Advances* online.

## 1 Introduction

Omics datasets, such as transcriptomics or phospho-proteomics, provide unbiased high-dimensional molecular profiles. However, their big dimensionality, combined with the highly connected nature of the molecules that are measured, makes it difficult to interpret them in a mechanistically relevant manner. Leveraging prior knowledge, we can use computational methods to infer which biological activities are relevant. For example, the activity of transcription factors (TFs) and kinases can be inferred robustly from downstream transcripts and phosphosite targets, respectively (Dugourd and Saez-Rodriguez, 2019). Over the past decade, a plethora of methods that infer biological activity has emerged, each with its own assumptions and biases.

Although comparisons and collections of these methods exist (Alhamdoosh *et al.*, 2017; Geistlinger *et al.*, 2016; Väremo *et al.*, 2013; Supplementary Table S1), they do not incorporate recent methodological developments, such as modeling activities based on weighted mode of regulation (Supplementary Table S2). Here, we present decoupleR, an R and Python package containing a collection of methods adapted for biological activity estimation in bulk, single-cell and spatial omics data.

## 2 Implementation

**Fig. 1.**
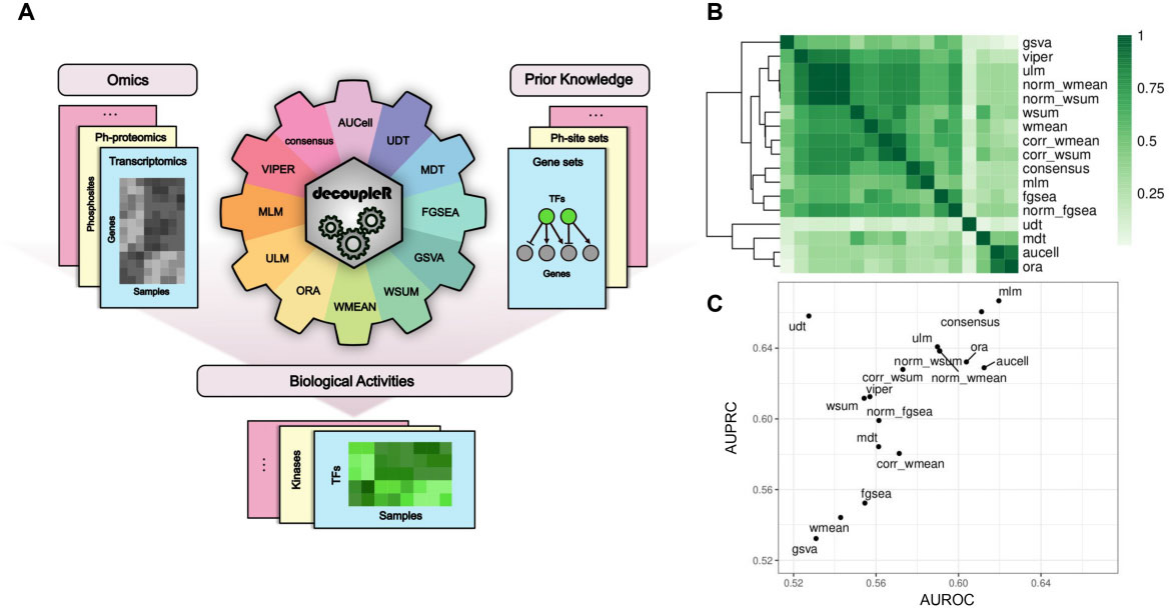
Inference of biological activities with decoupleR’s workflow. (**A**) decoupleR’s workflow, it contains a collection of computational methods that coupled with prior knowledge resources estimates biological activities from omics data molecular readouts such as normalized counts or log fold changes. (**B**) Spearman correlation across methods and (**C**) predictive performance across methods in the RNA-seq data-set

Currently, decoupleR contains 11 different methods (Fig. 1A), these include popular methods such as AUCell (Aibar *et al.*, 2017), fast GSEA (Korotkevich *et al.*, 2021), GSVA (Hänzelmann *et al.*, 2013), over-representation analysis, univariate linear model (ULM) adapted from Teschendorff and Wang (2020), VIPER (Alvarez *et al.*, 2016) and others (Supplementary Table S1). The inputs of decoupleR are: (i) a matrix containing molecular feature values, either for single samples or from population comparisons, like normalized gene expression counts per sample or log fold changes and (ii) a prior knowledge resource such as a collection of gene sets. The user can then choose any method alone or many simultaneously. decoupleR also provides a consensus score obtained by computing a mean z-score across methods (Supplementary Note). Additionally, decoupleR offers easy to use wrappers to query the meta-database OmniPath (Türei *et al.*, 2021), making it easy to flexibly access processed resources such as cell-type marker databases, gene regulatory networks or pathway footprints, and estimate biological activities from them.

## 3 Benchmark design

We used decoupleR to evaluate the performance of individual methods by recovering perturbed regulators—TFs and kinases—from two independent collections of transcriptomics (Holland *et al.*, 2020) and phospho-proteomics (Hernandez-Armenta *et al.*, 2017) datasets (Supplementary Note), respectively, upon single-gene perturbation experiments. As resources, we used the gene regulatory network DoRothEA (Garcia-Alonso *et al.*, 2019) and a kinase substrate network (Hernandez-Armenta *et al.*, 2017), respectively.

We built a benchmarking pipeline with decoupleR (Supplementary Note), which evaluates the performance of regulator activity scores from different methods, mainly focused on the sensitivity of methods. Furthermore, to evaluate the robustness of the methods to noise, we added or deleted a percentage of edges from the prior knowledge resources.

## 4 Results

Methods return different distributions of activities (Supplementary Fig. S1) but display general similarities (Supplementary Fig. S2), with a median Spearman correlation of activities between methods of 0.52, and 0.65 for transcriptomics and phospho-proteomics, respectively (Fig. 1B). There was also a moderate agreement between methods in the top 5% ranked regulators (median Jaccard indexes of 0.23 and 0.21, respectively; Supplementary Fig. S2).

Despite these similarities, methods showed different performances at predicting perturbed regulators (Supplementary Fig. S3). Some of them performed consistently better than the others (Supplementary Table S3; Fig. 1C), the top three being: consensus, multivariate linear model and ULM. Moreover, methods that leverage weights perform better when those are taken into account (*P*-value <2.2e-16; one-sided Wilcoxon signed-rank test; Supplementary Fig. S4).

Deleting edges in the resource had a greater effect than adding them across methods (Supplementary Fig. S5); with a median Spearman correlation of activities to the original ones of 0.84 and 0.77 for the addition and deletion, respectively (*P*-value <2.2e-16; one-sided Wilcoxon signed-rank test). Additionally, adding or deleting edges decreased predictability, and deleting edges had a worse effect than adding (adjusted *P*-values <2.2e-16 for normal-addition, <2.2e-16 for normal-deletion and <2.2e-16 for deletion-addition; *F* = 131; Tukey’s HSD *posthoc* test) (Supplementary Fig. S6).

Finally, we evaluated decoupleR’s speed and found that methods run relatively fast in the R version, and orders of magnitude faster in the Python one [median across methods of 1.44 and 0.44 ms per sample and regulator in R and Python, respectively, with an Intel(R) Core(TM) i7-8550U CPU @ 1.80 GHz; Supplementary Fig. S7], enabling their use with larger datasets such as single-cell or spatial omics.

## 5 Conclusion

In summary, decoupleR combines a variety of methods to infer biological activities into one efficient, robust, and user-friendly tool in the two most used programming languages for omics data analysis. With a common syntax for different methods, types of omics datasets, and knowledge sources available via OmniPath, it facilitates the exploration of different approaches and can be integrated in many workflows.

We observed that the majority of methods return adequate estimates of regulator activities, but that their aggregation into a consensus score and linear models perform better than other methods. We welcome the addition of further methods by the community.

## Supplementary Material

vbac016_Supplementary_Data

## References

[vbac016-B1] Aibar S. et al (2017) Scenic: single-cell regulatory network inference and clustering. Nat. Methods, 14, 1083–1086. 28991892 10.1038/nmeth.4463 PMC5937676

[vbac016-B2] Alhamdoosh M. et al (2017) Combining multiple tools outperforms individual methods in gene set enrichment analyses. Bioinformatics, 33, 414–424. 27694195 10.1093/bioinformatics/btw623 PMC5408797

[vbac016-B3] Alvarez M.J. et al (2016) Functional characterization of somatic mutations in cancer using network-based inference of protein activity. Nat. Genet., 48, 838–847. 27322546 10.1038/ng.3593 PMC5040167

[vbac016-B4] Dugourd A. , Saez-Rodriguez J. (2019) Footprint-based functional analysis of multiomic data. Curr. Opin. Syst. Biol., 15, 82–90. 32685770 10.1016/j.coisb.2019.04.002 PMC7357600

[vbac016-B5] Garcia-Alonso L. et al (2019) Benchmark and integration of resources for the estimation of human transcription factor activities. Genome Res., 29, 1363–1375. 31340985 10.1101/gr.240663.118 PMC6673718

[vbac016-B6] Geistlinger L. et al (2016) Bioconductor’s enrichment browser: seamless navigation through combined results of set- & network-based enrichment analysis. BMC Bioinformatics, 17, 45. 26791995 10.1186/s12859-016-0884-1 PMC4721010

[vbac016-B7] Hänzelmann S. et al (2013) GSVA: gene set variation analysis for microarray and RNA-seq data. BMC Bioinformatics, 14, 7. 23323831 10.1186/1471-2105-14-7 PMC3618321

[vbac016-B8] Hernandez-Armenta C. et al (2017) Benchmarking substrate-based kinase activity inference using phosphoproteomic data. Bioinformatics, 33, 1845–1851. 28200105 10.1093/bioinformatics/btx082 PMC5870625

[vbac016-B9] Holland C.H. et al (2020) Robustness and applicability of transcription factor and pathway analysis tools on single-cell RNA-seq data. Genome Biol., 21, 36. 32051003 10.1186/s13059-020-1949-z PMC7017576

[vbac016-B10] Korotkevich G. et al (2021) Fast gene set enrichment analysis. *bioRxiv.* DOI: https://doi.org/10.1101/060012.

[vbac016-B11] Teschendorff A.E. , Wang N. (2020) Improved detection of tumor suppressor events in single-cell RNA-seq data. NPJ Genomic Med., 5, 43. 10.1038/s41525-020-00151-y PMC7541488 33083012

[vbac016-B12] Türei D. et al (2021) Integrated intra- and intercellular signaling knowledge for multicellular omics analysis. Mol. Syst. Biol., 17, e9923. 33749993 10.15252/msb.20209923 PMC7983032

[vbac016-B13] Väremo L. et al (2013) Enriching the gene set analysis of genome-wide data by incorporating directionality of gene expression and combining statistical hypotheses and methods. Nucleic Acids Res., 41, 4378–4391. 23444143 10.1093/nar/gkt111 PMC3632109

