# Supplementary material for: decoupleR: ensemble of computational methods to infer biological activities from omics data
Source: Bioinform Adv. 2022 Mar 8;2(1):vbac016. doi: 10.1093/bioadv/vbac016 (PMC9710656; doi:10.1093/bioadv/vbac016)
Supplement: vbac016_Supplementary_Data [file vbac016_supplementary_data.pdf]

Supplementary Information:

Methods overview

AUCell

AUCell (Aibar *et al.*, 2017) uses the Area Under the Curve (AUC) to calculate whether a set of targets is enriched within the molecular readouts of each sample. To do so, AUCell first ranks the molecular features of each sample from highest to lowest value, resolving ties randomly. Then, an AUC can be calculated using by default the top 5% molecular features in the ranking. Therefore, this metric represents the proportion of abundant molecular features in the target set, and their relative abundance value compared to the other features within the sample.

Univariate Decision Tree

Univariate Decision Tree (UDT) fits a single decision tree for each regulator and sample. As a unique covariable, UDT uses the associated weights of a given regulator to estimate the molecular readouts of all molecular features in a sample. Target features with no associated weight are set to zero. The obtained feature importance from the fitted model is the activity of the regulator.

Multivariate Decision Trees

Multivariate Decision Trees (MDT) fits an ensemble of decision trees, known as random forest, to infer regulator activities. MDT, contrary to UDT, uses all regulators of a given network to estimate the molecular readouts of all molecular features in a sample. Same as UDT, target features with no associated weight are set to zero. The feature importances extracted from the fitted model are the regulator activities.

Fast Gene Set Enrichment Analysis

Fast Gene Set Enrichment Analysis (FGSEA) (Sergushichev, 2016) estimates regulator activities using a GSEA implementation based on an adaptive multi-level split Monte Carlo scheme. In GSEA, molecular features are first ranked per sample. Then, an enrichment score (ES) is calculated by walking down the list of features, increasing a running-sum statistic when a feature in the target feature set is encountered and decreasing it when it is not. The magnitude of the increment depends on the correlation of the molecular feature with the regulator being evaluated. The final ES is the maximum deviation from zero encountered in the random walk. Finally, a normalized ES (NES), called *norm_fgsea* in decoupleR, can be calculated using permutations.

Gene Set Variation Analysis

Gene Set Variation Analysis (GSVA) (Hänzelmann *et al.*, 2013) starts by transforming the input molecular readouts matrix to a readout-level statistic using Gaussian kernel estimation of the cumulative density function. Then, readout-level statistics are ranked per sample and normalized to up-weight the two tails of the rank distribution. Afterwards, an enrichment score (ES) is calculated as in GSEA, using the running sum statistic. Finally, the ES can be normalized by subtracting the largest negative ES from the largest positive ES.

Weighted Sum

Weighted Sum (WSUM) infers regulator activities by first multiplying each target feature by its associated weight which then are summed to a final enrichment score (ES). It can be defined as:

$$ES = \sum_{i=1}^{n} s_i l_i X_i$$

Where n is the number of targets for a given regulator, s_i is the associated mode of regulation (either positive of negative), l_i is the likelihood of that event happening and X_i is a molecular feature statistics like gene expression. In case s_i or l_i are not present, these are set to one.

Furthermore, permutations of random target features can be performed to obtain a normalized score (NES), called *norm_wsum* in decoupleR, with R being the obtained random null distribution:

$$NES = \frac{ES - mean(R)}{sd(R)}$$

A corrected enrichment score (CES), called *corr_wsum*, is also obtained:

$$CES = -log10(p) * ES$$

Where p is the empirical p-value defined as:

$$p = \frac{r}{N}$$

$$if\, p = 0,\ p = \frac{1}{N}$$

$$if\, p = 1,\ p = \frac{N-1}{N}$$

Here, r is the number of times R was bigger than the absolute value of ES and N is the number of random permutations. NES and CES are alike, but CES can handle better zero inflated distributions since NES requires a high N value to avoid having a $sd(R)$ equal to zero.

Weighted Mean

Weighted Mean (WMEAN) is similar to WSUM but it divides the obtained ES by the sum of the absolute value of weights. It can be defined as:

$$ES = \frac{\sum_{i=1}^{n} s_i l_i X_i}{\sum_{i=1}^{n} abs(s_i l_i)}$$

Like in WSUM, a NES (*norm_wmean*) and a CES (*corr_mean*) can be calculated if random permutations of target features are performed. It is worth mentioning that *norm_wmean* and *norm_wsum* converge into the same scores since their null distributions are the same.

Over Representation Analysis

Over Representation Analysis (ORA) measures the overlap between the target feature set and a list of most altered molecular features in the input matrix. The most altered molecular features can be selected from the top and/or bottom of the molecular readout distribution. ORA first builds a contingency table and then runs a one-tailed Fisher's exact test to determine if a regulator's set of features are enriched in the selected features from the data. The resulting score is:

$$ES = -log_{10}(p)$$

Where p is the obtained p-value from the test.

Univariate Linear Model

Univariate Linear Model (ULM), adapted from (Teschendorff and Wang, 2020), like UDT, uses as a unique covariable the weighted mode of regulation of a single regulator to estimate the molecular readouts of all molecular features in a sample. Target features with no associated weight are set to zero. The obtained t-value from the fitted model is the activity of the regulator.

Multivariate Linear Model

Multivariate Linear Model (MLM), contrary to ULM and similar to MDT, uses all regulators of a given network to estimate the molecular readouts of all molecular features in a sample. Same as ULM, target features with no associated weight are set to zero and the obtained t-values from the fitted model are the activities of the regulators.

VIPER

Virtual Inference of Protein-activity by Enriched Regulon analysis (VIPER) (Alvarez *et al.*, 2016) estimates biological activities by performing a three-tailed enrichment score calculation. First, a ranking is performed for the absolute value of the molecular statistics in the input matrix per sample. The closer value to zero in the matrix is given a ranking of one and the most extreme positive value is given a ranking of N. Then, these rankings are quantile transformed. The one-tailed enrichment score is computed as:

$$ES_1 = \frac{\sum_{i=1}^{n}(1-abs(s_i))l_iK_i}{n}$$

Here, n is the number of targets for a given regulator, s_i is the associated mode of regulation, l_i is the likelihood of that interaction and K_i is the quantile-transformed ranking of molecular statistics. Next, molecular targets inside each regulator are ranked again, now based on the mode of regulation, either positive or negative:

$$Q = rank(S * X)$$

S is a vector indicating the mode of regulation for each target feature and X is a vector containing the molecular statistics from a given sample. Ranks are also quantile transformed and the two-tailed ES is calculated as:

$$ES_2 = \frac{\sum_{i=1}^{n} s_i l_i Q_i}{n}$$

Q_i is the two-tailed quantile-transformed ranking of molecular statistics. Then, the three-tail score is defined as:

$$ES = (|ES_2| + ES_1) \times s$$

Where s is the sign of ES_2. Finally a normalized enrichment score is estimated by:

$$NES = ES * \sqrt{\sum_{i=1}^{n} l_i^2}$$

Which is an analytical approximation to random permutations.

Consensus

A consensus score is generated when more than one method is run with decoupleR. For each method, the obtained activities are transformed into z-scores, first for positive values and then for negative ones. These two sets of z-score transformed activities are computed by subsetting the values bigger or lower than 0, then by mirroring the selected values into their opposite sign and finally calculating a classic z-score. This transformation ensures that values across methods are comparable, and that they remain in their original sign (active or inactive). The final consensus score is the mean across different methods.

Benchmark design

We used decoupleR to evaluate the performance of individual methods by recovering perturbed transcription factors (TFs) from a curation of single-gene perturbation experiments (Holland *et al.*, 2020). As a resource we used DoRothEA, a gene regulatory network linking TFs to target genes by their mode of regulation (Garcia-Alonso *et al.*, 2019). Perturbation experiments where the targeted regulator

was not in DoRothEA were removed. After filtering, this dataset is composed of gene expression data from 92 knockdown and overexpression experiments of 40 unique TFs in human cells. Additionally, we tested the performance of decoupleR on phospho-proteomic data. For this, we filtered in a similar fashion a curated set of knockdown and overexpression single-kinase perturbation experiments, obtaining 63 experiments including 14 unique kinases, and applied a weighted resource from the same publication that links kinases to their target phosphosites (Hernandez-Armenta et al., 2017). For the transcriptomic dataset, differential expression analysis was performed with limma and the resulting t-values were used as input. For the phospho-proteomics, the quantile-normalized log2-fold changes from different studies were used to make them comparable. The unprocessed data can be accessed through Zenodo: https://zenodo.org/record/5645208.

We built a benchmarking package using decoupleR, called decoupleRBench (https://github.com/saezlab/decoupleRBench) which evaluates the performance of TF and kinase activity scores from different methods. Regulator activities were inferred from perturbation experiment data for both omics datasets using every method with default parameters. Since we only have one perturbed regulator for each experiment, we decided to concatenate all experiments into a single vector to have more than one True Positive case. Afterwards, we transformed the obtained scores to their absolute value. Since there are overexpression and knockout perturbation experiments, we assumed that perturbed regulators can have either highly positive or highly negative scores. Moreover, given that the true positive classes are limited by the TFs or kinases covered in the perturbation experiments, we added a downsampling strategy, where for each permutation an equal number of negative classes was randomly sampled. Finally, the area under the Receiver operating characteristic (AUROC) and Precision Recall curve metrics (AUPRC) were computed for each downsampling permutation. For the phospho-proteomics dataset, we ran two versions of the prior knowledge resource, one without weights and one with weights coming from kinase binding potentials, to assess whether the addition of weights gave any additional value to the prediction precision.

The obtained activities were further compared by computing the Spearman

correlation between the concatenated scores of all samples from one method to another. We also checked the overlap of regulators with high absolute value score between methods by computing the Jaccard index of each pair of experiments. The final Jaccard index comes from calculating the median across experiments.

Furthermore, to evaluate the robustness of the methods to noise, we added or deleted a percentage of edges (25%, 50% and 75%) to every regulator in the prior knowledge networks. When random edges were added, their mode of regulation and weight were set to 1. For every mode (addition or deletion) and percentage, we generated five different networks, which we ran through the benchmarking pipeline of decoupleRBench. With the inferred regulator scores, for every percentage and mode we measured robustness as the correlation of scores with the normal ones and the difference of performance in AUROC and AUPRC to the normal networks.

Supplementary tables

Supplementary Table 1. Comparison of methods available across different packages.

	Packages			
Methods	PIANO	Enrichment Browser	EGSEA	decoupleR
AUCell				X
UDT				X
MDT				X
GSEA	X	X	X	X
GSVA		X	X	X
WSUM				X
WMEAN				X
ORA		X	X	X
ULM				X
MLM				X
VIPER				X

Supplementary Table 2. List of methods currently available in decoupleR. Methods are classified by whether they model the mode of regulation and the weight of the source-target link, whether they are based on permutations, whether they generate a p-value associated with the inferred score and by their range of values.

Name	Citation	Weight	Permutation	p-value	Range
AUCell	(Aibar *et al.*, 2017)	No	No	No	0,1
UDT		Yes	No	No	0, Inf
MDT		Yes	Yes	No	0, Inf
FGSEA	(Korotkevich *et al.*, 2016)	No	Yes	Yes	-Inf, +Inf
GSVA	(Hänzelmann *et al.*, 2013)	No	No	No	-1, +1
WSUM	-	Yes	Yes	Yes	-Inf, +Inf
WMEAN	-	Yes	Yes	Yes	-Inf, +Inf
ORA		No	No	Yes	0, Inf
ULM	Adapted from (Teschendorff and Wang, 2020)	Yes	No	Yes	-Inf, +Inf
MLM		Yes	No	Yes	-Inf, +Inf
VIPER	(Alvarez *et al.*, 2016)	Yes	No	Yes	-Inf, +Inf
Consensus		Yes	No	Yes	-Inf, Inf

Supplementary Table 3. List of methods ranked by their performance in the benchmarking pipeline. Methods are ranked by the median area under the curve (AUC) of the joint distribution of all downsampling permutations in both AUROCs and AUPRCs for both datasets. Methods with significant p-values have a greater distribution of AUCs than the rest, computed using the one-sided Mann-Whitney U test (N=6.40e+05).

Method	p-value	Median AUC
consensus	<2.2e-16	0.67
mlm	<2.2e-16	0.67
ulm	<2.2e-16	0.66
norm_wmean/norm_wsum	<2.2e-16	0.65
ora	<2.2e-16	0.64
corr_wsum	<2.2e-16	0.64
mdt	<2.2e-16	0.62
wsum	0.144	0.64
viper	1	0.62
aucell	1	0.62
corr_wmean	1	0.62
wmean	1	0.60
fgsea	1	0.59
norm_fgsea	1	0.58
gsva	1	0.56
udt	1	0.54

Supplementary figures

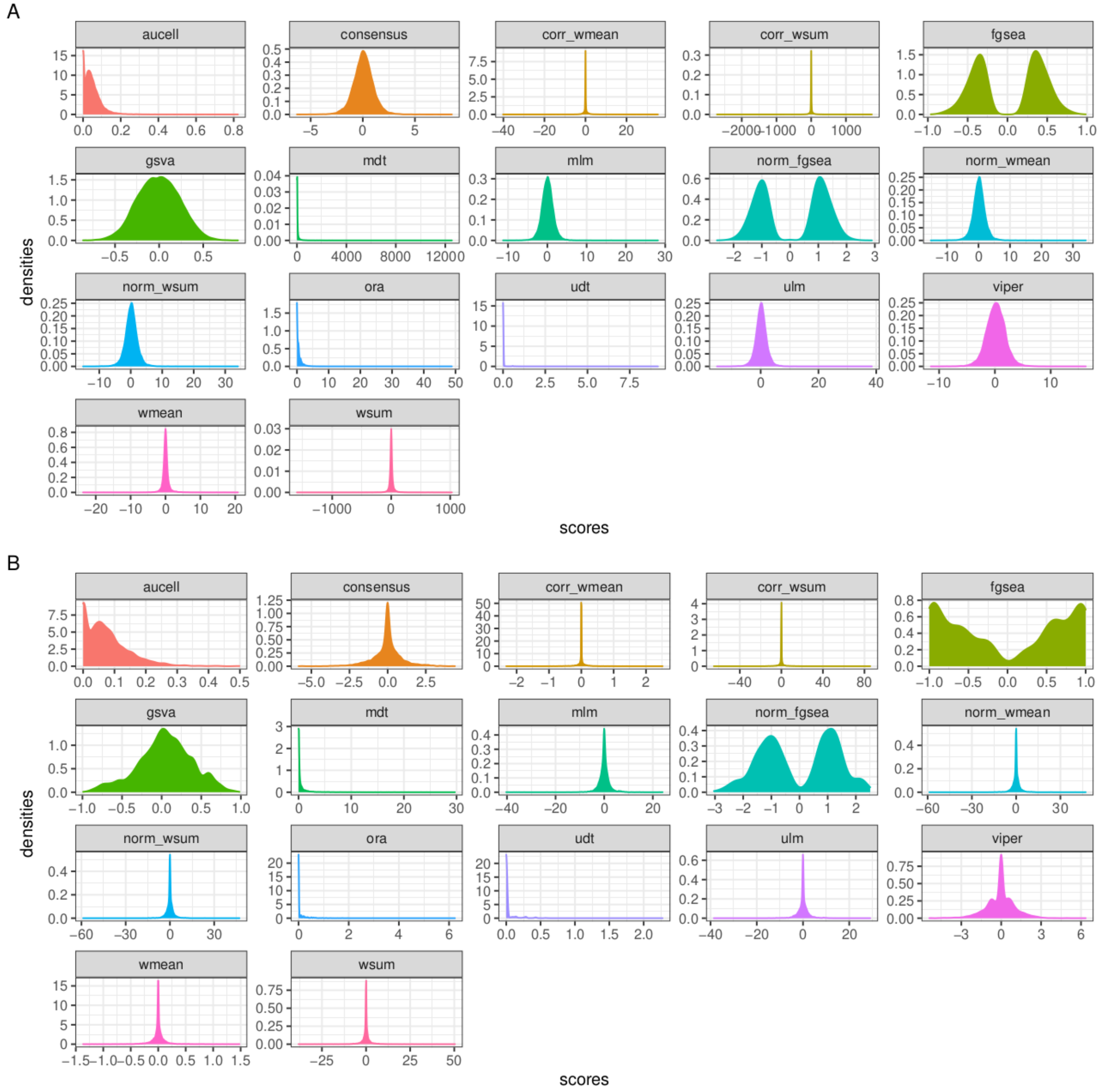

Supplementary Figure 1. Method scores distributions for the transcriptomic dataset (A) and phospho-proteomics dataset (B).

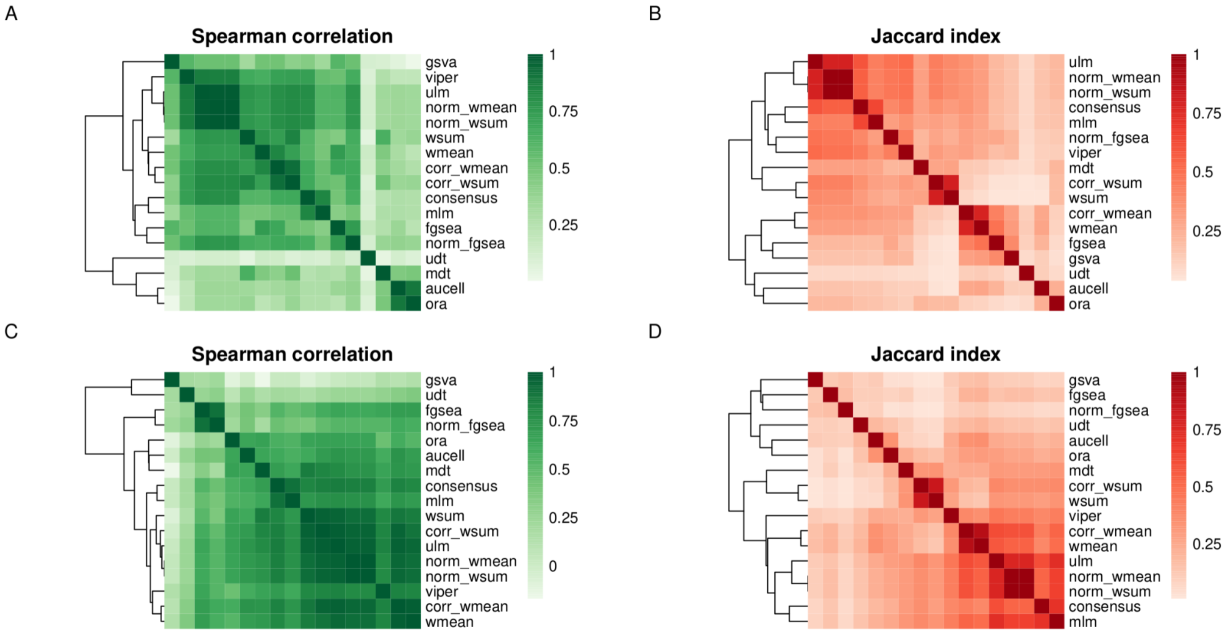

Supplementary Figure 2. Spearman correlations between methods using the transcriptomics (A) and phospho-proteomics (C) datasets. Median Jaccard index between methods of the top 5% TFs (B) or kinases (D) ranked by the absolute value of enrichment score.

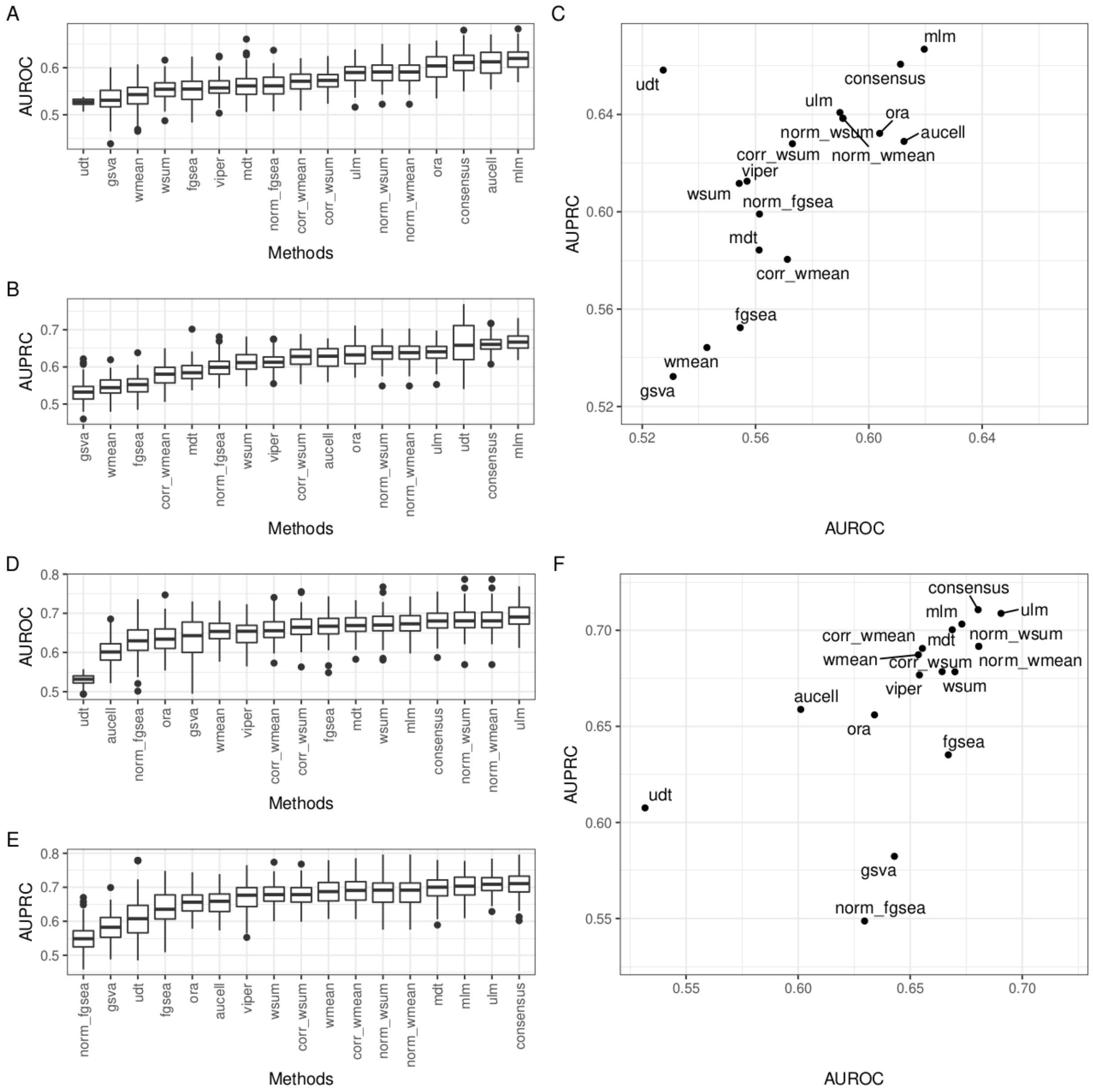

Supplementary Figure 3. Distributions of AUROCs (A), AUPRCs (B) and the median for both (C) for each method in the transcriptomics dataset. Distributions of AUROCs (D), AUPRCs (E) and the median for both (F) for each method in the phospho-proteomics dataset.

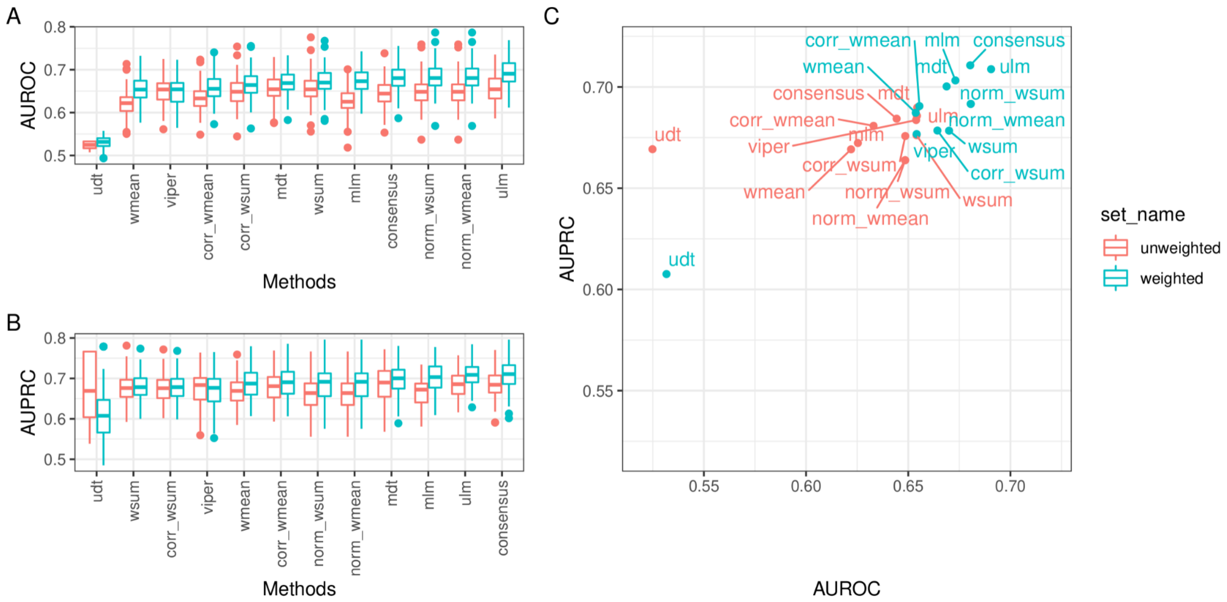

Supplementary Figure 4. Distributions of AUROCs (A), AUPRCs (B) and the median for both (C) for each method in the phospho-proteomics dataset. Color indicates if the weights of the prior knowledge resource were used.

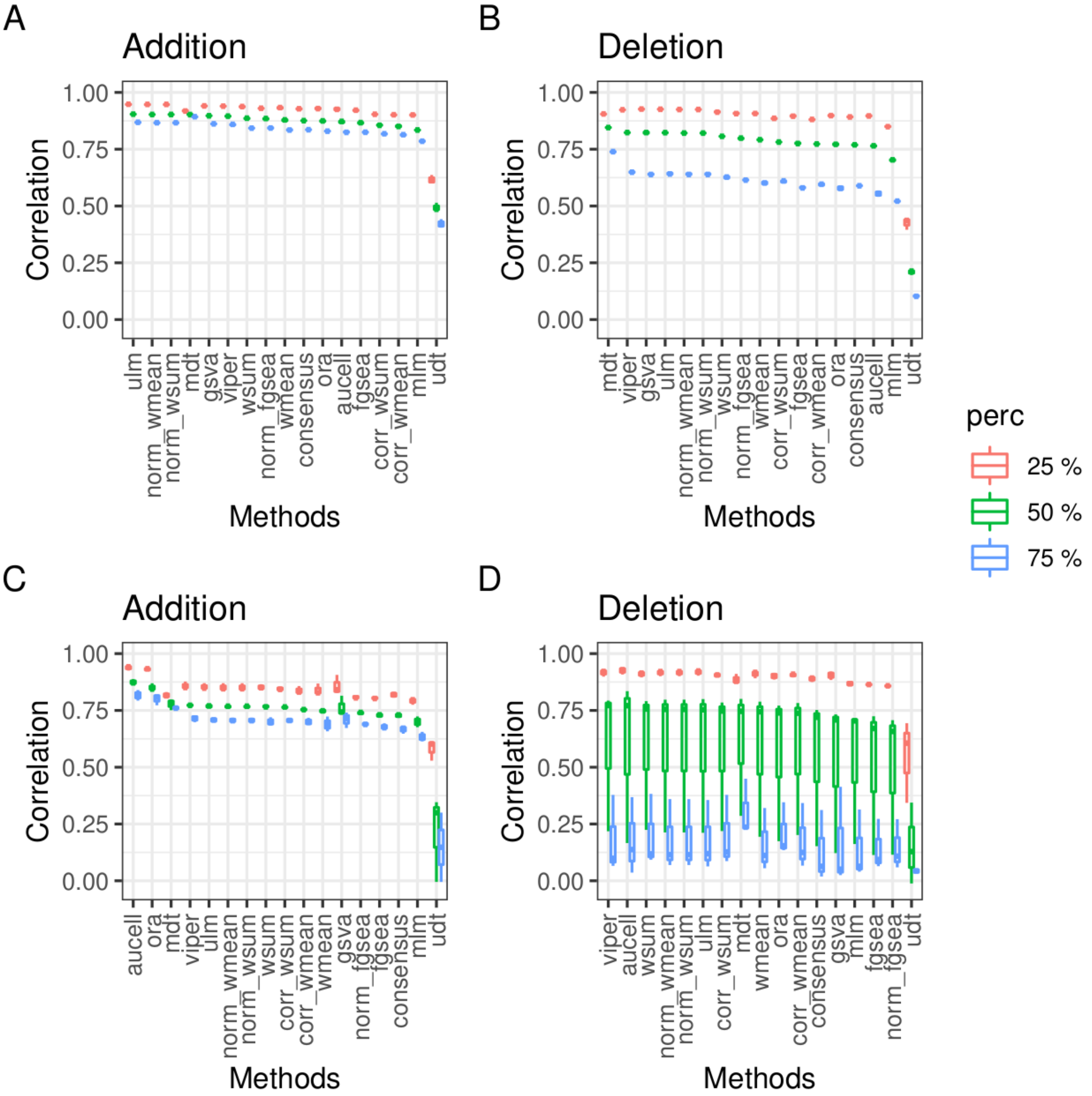

Supplementary Figure 5. Correlations between original enrichment scores and scores obtained after adding or deleting a percentage of edges to the prior knowledge resource used for the transcriptomic (A,B) and phospho-proteomic (C,D) datasets.

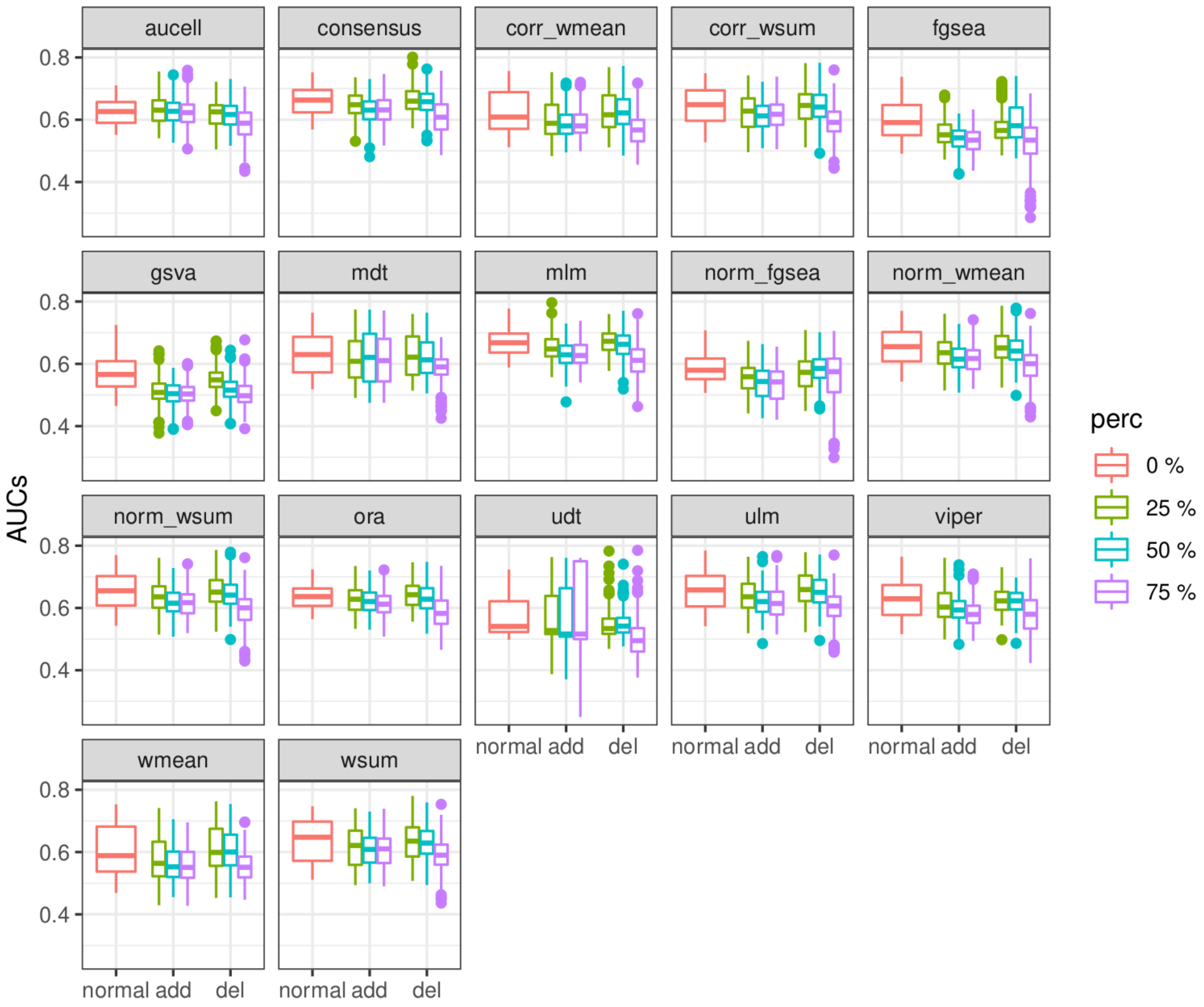

Supplementary Figure 6. Distributions of AUROCs and AUPRCs for both datasets obtained after adding or deleting edges in the prior knowledge resource.

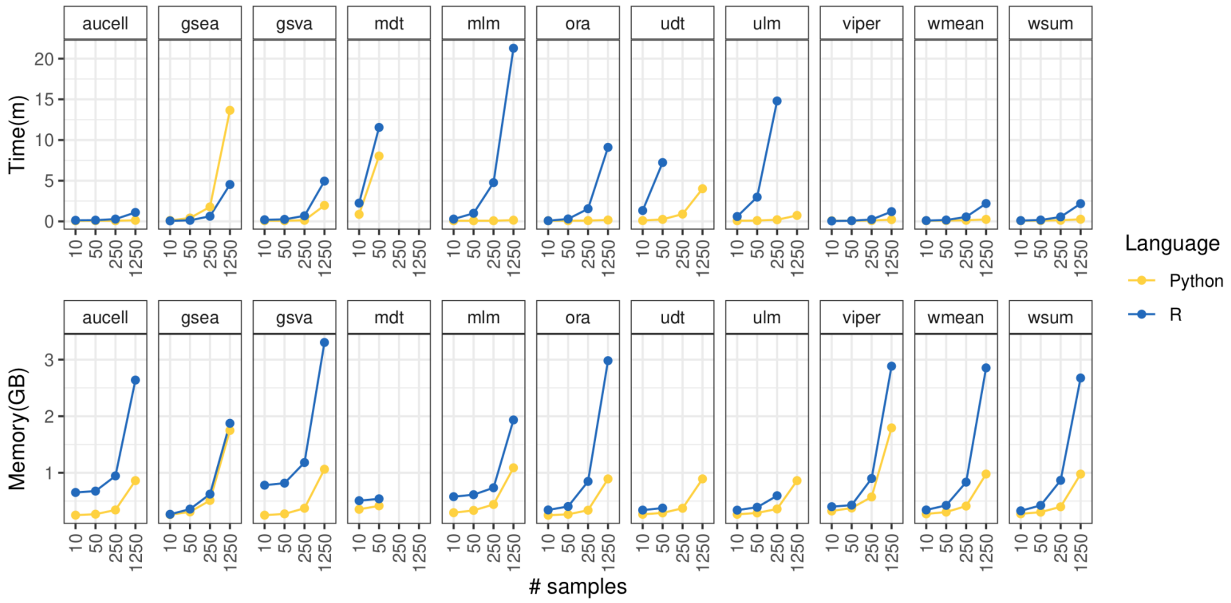

Supplementary Figure 7. Runtime and memory consumption across programming languages for each method, using as input a network containing 250 sources and a matrix with 20.000 targets with increasing numbers of samples.
